# Effects of abiotic environmental factors and land use on the diversity of carrion-visiting silphid beetles (Coleoptera: Silphidae): A large scale carrion study

**DOI:** 10.1371/journal.pone.0196839

**Published:** 2018-05-30

**Authors:** Christian von Hoermann, Dennis Jauch, Carolin Kubotsch, Kirsten Reichel-Jung, Sandra Steiger, Manfred Ayasse

**Affiliations:** 1 Institute of Evolutionary Ecology and Conservation Genomics, University of Ulm, Ulm, Germany; 2 Department of Conservation and Research, Bavarian Forest National Park, Grafenau, Germany; Universidade Federal de Goias, BRAZIL

## Abstract

Anthropogenic land use causes global declines in biodiversity. Despite the knowledge that animal carrion is the most nutrient-rich form of dead organic matter, studies on landscape and local scales determining whether and the means by which land use intensity influences the diversity of the carrion-associated insect fauna are globally scarce. We investigated the effects of land use intensity and abiotic and biotic environmental factors on the abundance, species richness, and diversity of the important ecosystem-service-providing silphid beetle taxon (carrion beetles) in three regions of Germany. In 61 forest stands distributed over three geographically distinct regions in Central Europe, we trapped silphid beetles on exposed piglet cadavers during late summer. In all three regions, higher ambient temperatures and higher fine sand contents were associated with the abundance of the silphid beetle taxa. The carrion community silphid diversity was negatively affected by an increase in mean ambient temperature in all three regions. Although management intensity in forests did not affect the overall abundance of Silphidae, the abundance of *Nicrophorus humator* decreased significantly with higher forest management intensity across all three regions. Unmanaged and age-class forests showed a higher abundance of *N*. *humator* compared with extensively managed forest stands. These findings indicate that *N*. *humator* has potential as an indicator species for anthropogenic disturbances in forests. Overall, the direct responses of the silphid beetle community to diverse soil characteristics underline soil as an important factor determining the abundance and diversity of necrophagous carrion beetles in Central Europe. To protect these valuable ecosystem-service providers, forest-management-induced soil modifications need to be paid close attention.

## Introduction

Increasing land use intensity and land use change are major drivers of biodiversity loss, particularly in forest ecosystems [1–3]. Approximately 82% of Central European forests are human-dominated and therefore are highly disturbed [4]. In many forests, intensified age-class forestry has reduced the quality of the habitat and also its structural heterogeneity [5]. The homogenization of such anthropogenically influenced ecosystems on the landscape scale, whereby species assemblages become increasingly dominated by a small number of widespread species, is one of the main threats to biodiversity [6,7]. Commendably, in recent years, modern forest management strategies have avoided large-scale clear-felling in age-class forests or have established increased amounts of dead wood in forests to increase species richness [8]. Nevertheless, the above indicates that land use type and intensity affects the diversity of insects, including that of forest-dwelling carrion insect communities; this in turn might have a negative impact on the important ecosystem services, such as carcass removal rate and nutrient cycling, carried out by these insects [9,10].

Animal carrion is the most nutrient-rich form of dead organic matter and decomposes at a fast rate [11–13]. These two key qualities of high nutrient concentration and accelerated temporal dynamics make carrion a highly important component of the detritus pool [14]. Carrion has a significant impact on terrestrial biodiversity and ecosystem properties through its influence on below-ground microbial communities, soil nutrients, arthropods and on scavenging vertebrates [15]. Consequently, animal carrion is a precondition for the evolution and maintenance of detritivore and decomposer diversity, and in turn, the diversity of detritivores and decomposers impacts nutrient cycling rates and ultimately influences producer and consumer diversity [12].

In terrestrial ecosystems, the decomposition and dispersion of carrion nutrients is heavily dependent on abiotic factors, such as the temperature, humidity, soil type, and pH-values of soil (e.g., see references in [16]), and on the availability of insect detritivores and decomposing microorganisms [17]. Consequently, for the continuous functioning of ecosystem processes and services, the biodiversity of the carcass-associated insect and microbial fauna must be preserved, and therefore, their influencing factors need to be identified.

For functional arthropod groups such as predators and wood decomposers, several studies have found clear indications that they are negatively affected by forest management (e.g., [18,19]). Beetles (Insecta: Coleoptera) occupy diverse niches, and several species are specific to their given substrates (e.g., [20,21]). Considering these aspects, beetles involved in the process of decomposition will often form a significant part of the biodiversity of their carrion microhabitat [21–23]. In particular, carrion beetles (Coleoptera: Silphidae) are frequently associated with vertebrate cadavers and provide a wide range of ecosystem services [24,25] by promoting the breakdown and recycling of organic matter into terrestrial ecosystems [26–29]. Most silphid species are necrophagous but can also prey on carrion-inhabiting necrophagous fly larvae, other small necrophilous carrion beetles, and fly eggs [26,27,30,31]. The taxon Silphidae is part of the taxon Staphylinoidea and is divided into two groups: the Nicrophorinae (11 species in north western Europe; all from the genus *Nicrophorus*, called burying beetles) and the Silphinae (17 species in north western Europe) [24,26,30,32,33,34]. According to their name, burying beetles (*Nicrophorus*) bury small vertebrate cadavers in the soil as food for their larvae [35]. The elaborate biparental care carried out by one conspecific pair of beetles, which have secured a freshly dead cadaver suitable for reproduction, has been known for a long time in the taxon *Nicrophorus* [36]. Burying beetles also colonize large vertebrate cadavers in high numbers [37,38]. Dozens of burying beetle individuals, particularly during the period when their ovaries are maturing, converge on large cadavers that are too large for burial and use them as feeding sites (> 300 g, [24,36]). In contrast to the Nicrophorinae, female Silphinae species are semelparous and lay their eggs in or on the soil around large vertebrate cadavers, and no parental care of their larvae is provided [24,30,39]. Silphids promote the recycling of nutrients and their necrophagous feeding activities may also destroy some foci of infection of human pathogenic bacteria [40].

Dynamic changes in the composition of organisms (especially arthropods) that visit carrion during its various decomposition stages has been widely documented [41–45]. However, as no large-scale carrion study is available that explicitly examines the interacting effect of land use intensification and biotic and abiotic environmental factors on overall carrion ecology [17], key knowledge gaps still exist concerning the effect of land use on carcass-inhabiting insect diversity, species richness and abundance, and consequently, their critical ecosystem services. To address this area of knowledge, we have conducted a large-scale study in which we have exposed 75 piglet cadavers across differently managed forest stands in Central Europe and monitored cadaver-visiting silphid beetles during the whole course of decomposition. We have hypothesised that forest management intensity and other biotic and abiotic environmental factors will affect silphid beetle abundance, species richness, and diversity. Forest management intensity has been quantified by using the recently developed silvicultural management intensity indicator (SMI), which combines three main characteristics of a given stand: stand age, tree species, and aboveground, living, and dead wooden biomass [46]. Our conclusions can be generalized because our study encompasses three regions differing in geology, topography, and climate.

## Methods and materials

### Ethics statement

All necessary permits were obtained for the described field studies. No animals were killed for this study. All cadavers of exclusively stillborn piglets were obtained under veterinary supervision (special permit for animal by-products (EG) No. 1069/2009) from a local pig farmer (Winfried Walter, Gögglingen, Germany). For field sampling of arthropods, an exemption existed concerning § 67 BNatSchG and species protection legislation according to § 45 BNatSchG.

### Study sites and piglet cadaver exposure

We conducted our study in three different geographical regions in Germany as specified by the framework of the Biodiversity Exploratories (http://www.biodiversity-exploratories.de): the Schwäbische Alb (Baden-Württemberg, 48° 20´ 60.0´´ N to 48° 32´ 3.7´´ N; 9° 12´ 13.0´´ E to 9° 34´ 48.9´´ E) in the South-West, the Hainich-Dün region (Thuringia, 50° 56´ 14.5´´ N to 51° 22´ 43.4´´ N; 10° 10´ 24.0´´ E to 10° 46´ 45.0´´ E) in Central Germany, and the Biosphere reserve Schorfheide-Chorin (Brandenburg, 52° 47´ 24.8´´ N to 53° 13´ 26.0´´ N; 13° 23´ 27´´ E to 14° 8´ 52.7´´ E) in the North-East. A more detailed description of the three regions is supplied in supplemental methods. In all, 75 forest experimental plots (EPs, 25 in each of the three regions) of one hectare each were selected following a stratified random design with strata representing diverse forest management intensities and several other abiotic factors such as soil type and soil depth (Fig A1 in S2 File, [47]). These 25 plots chosen per region represent the existing range of different land use intensities [47].

From August 4^th^ until September 4^th^ 2014, we simultaneously exposed 75 stillborn piglet cadavers (*Sus scrofa domestica*, 1.44 kg average weight) on 25 forest EPs per region (one piglet per plot, Fig A1 in S2 File). EPs were sufficiently spaced at a minimum distance of 200 m between the outer edges of two EPs (Biodiversity Exploratories criteria, after [47]) to avoid cross interactions among individual cadavers. We used piglets as a carrion substrate because of their well-studied and assured succession of carrion insects, and because they are a well-established model system in forensic entomology (e.g., [45,48,49,50]). Furthermore, they are present nationwide as the wild-type *Sus scrofa* (wild boar) in the forest habitats of Germany. After a defrosting period of 24 hours, freshly dead piglet exposure started on August 4^th^ (n = 38) and 5^th^ (n = 37) and lasted until September 3^th^ (n = 38) and 4^th^ (n = 37), respectively. All cadavers of exclusively stillborn piglets were obtained under veterinary supervision (special permit for animal by-products (EG) No. 1069/2009) from a local farmer in Gögglingen (Baden-Württemberg, Germany) and were frozen (-20 °C) up until 24 hrs before the start of exposure. Since the study aimed to focus on insect communities, all piglets were exposed in black wire cages (63 cm x 48 cm x 54 cm, MH Handel GmbH, Munich, Germany) to exclude feeding and removal by larger scavengers such as foxes, wild boars, or raccoons. We mounted data loggers (Thermochron iButton, Whitewater, WI, USA) inside of each wire cage to record the temperature of the carrion microhabitat every 30 minutes during the whole fieldwork period. Wire cages containing cadavers and controls (pitfall traps without carcasses and wire cages) were installed at a distance of 100 m to each other within differently managed forest stands (Fig A1 in S2 File). Controls were needed to capture the prevailing and not necessarily carrion-associated insect fauna of the habitat (Fig A1 in S2 File).

### Installation of pitfall traps and beetle sampling

On the periphery of each cadaver, we installed two pitfall traps for trapping of cadaver-associated insects. One pitfall trap was installed adjacent to the head of the piglet, with the other one being adjacent to its anus. This allowed us to situate both traps inside each wire cage by taking into consideration two important settlement areas (head and anus) for cadaver-inhabiting insects [51]. Pitfall traps were composed of two ground-level smoothie cups stacked inside each other (half-liter PLA cups; diameter: 95 mm, height: 151.2 mm; Huhtamaki Foodservice GmbH, Alf/Mosel, Germany). The inner cup was filled with an odorless soapy solution (one drop of detergent, Klar EcoSensitive, AlmaWin, Winterbach, Germany) to reduce surface tension. For protection against rainfall, each single trap was equipped with a rain cover (constructed at Ulm University, Ulm, Germany). For controls, we applied the same procedure as described above, with the only differences being no cadaver and no wire cage in these cases. For reasons of comparability, the distance of the two control traps at one single capture site corresponded to the distance between the piglet head and anus. A total of 7 trap-emptying events per exposed cadaver and control during the whole decomposition period were conducted: at 2, 4, 6, 9, 16, 23, and 30 days after day 0 of exposure. These sampling intervals covered all the distinct stages of decomposition based on large-scale succession data in the literature [45,49]. At 48 hrs before the trap-emptying events, we opened the lid covered the pitfall traps (PLA dome-covers for smoothie cups; diameter: 95 mm; Huhtamaki Foodservice GmbH, Alf/Mosel, Germany) to guarantee a constant sample period for each trapping event. Therefore, each insect sampling event lasted 48 hrs. For later morphological assessment and classification of decay stages in the laboratory [20], all of the conducted trap-emptying events were accompanied by photo-documentation of the decomposition stages of all exposed piglet cadavers.

All collected insect individuals were transferred into 70% ethanol (VWR International GmbH, Darmstadt, Germany) for later sorting to larger taxonomic groups and subsequent species identification in the laboratory. All silphid individuals were identified to species level [52] and stored at Ulm University (Institute of Evolutionary Ecology and Conservation Genomics, Department of Biology). For any single trap-emptying event, we pooled all data for the 2 cups on either side of each piglet on each plot. The same was true for the controls. Because of losses of piglet cadavers (one cadaver in the Schwäbische Alb and three cadavers in Hainich-Dün) and the prohibition of the right of entry on particular sampling days to a total of 10 plots, the sampling campaign resulted in 294 sample units for the Schwäbische Alb, 224 sample units for Hainich-Dün, and 336 sample units for Schorfheide-Chorin. All these 854 sample units from overall 61 plots formed the basis for later statistical analysis.

### Environmental variables

We considered a total of 21 biotic and abiotic environmental variables in our analyses. All variables and their respective values were known from several inventory campaigns carried out within the Biodiversity Exploratories (basic data including soil type, soil composition, bulk density (for the upper 10 cm of the mineral soil, units: g/cm^3^), climate, vertical structure, and management). Exemplarily, soil type and soil composition were considered as important abiotic environmental parameters in our analyses, because soil characteristics are known as an important factor determining the local abundance of carrion beetles [25,36,53].

### Statistical analyses

All analyses were conducted in R version 3.3.1 ([54], 2016). Kruskal-Wallis rank sum tests with post hoc pairwise comparisons by using Tukey tests were applied to test the effects of the decomposition stage and trap type (cadaver versus control) on overall silphid beetle abundance.

For the quantification of the relative importance of environmental variables on total silphid beetle abundance, species richness, and diversity, we used the random forest approach (randomForest function implemented in the MASS package) to identify those environmental variables with an increase of more than 50% of the mean square error—in the case of omission—together with the marked highest IncNodePurity-values out of all 20 variables considered in this study (Figures A1—A7 in S1 File, after [55,56] and [5]). The random forest approach is a recursive partitioning and classification tree method [57] based on regression trees by using random inputs [58,59].

Further, we used generalized linear mixed models (GLMMs) to test for any effects of the environmental variables on the total abundance, species richness, and diversity (Shannon’s diversity and Simpson’s dominance) of the silphid beetle taxon across differently managed forest stands. Such differences were investigated across all silphid beetle taxa and, in the case of total abundance, also separately for the single silphid species *Nicrophorus vespilloides*, *N*. *investigator*, and *N*. *humator*. Negative binomial error distributions were applied in all those models in which overdispersion was present when previously fitted with a Poisson error distribution (after [60]). Our data are expected to be temporally dependent within each plot, as they are collected across experimental plots during seven subsequent visits. Therefore, we fitted our regression models with a random effect at the plot level. Plot-specific random effects should capture most of the latent heterogeneity (and over-dispersion) of the data. We further investigated forest management intensity by using a precalculated index (SMI) that can be described by two components, risk of stand loss and stand density, which theoretically are independent of one another [46]. The risk component defines the combined effect of stand age and tree species selection on SMI [46]. The other component, stand density, quantifies the effect of removals and regeneration method using actual biomass related to a reference [46]. Schall and Ammer (2013) commented that SMI at the operational level is mostly related to fellings (tending, thinning and harvest operations), but in the case of trees remaining in the stand due to natural losses (e.g. windthrow), the discrepancy between fellings and removals becomes even more evident. They stated that removals (used for SMI description in the risk component) are more indicative of silvicultural management intensity than trees that are lost due to silvicultural or natural reasons [46]. Schall and Ammer (2013) consequently proposed to measure removals by the deviance between maximum biomass (species, age and site specific) and actual biomass of living and dead trees. We included all three single components ‘main tree species’, ‘stand density’ and ‘stand age’ as fixed factors in our GLMMs to test for effects on the respective response variables (abundance, species richness, Shannon’s diversity and Simpson’s dominance). The combined SMI-index, together with the fixed effect at the region level (variable *exploratory*) was considered in separate negative binomial-, gamma- or Gaussian-GLMs (the two last families were used to test the effects of forest management intensity and region on silphid diversity—after examining diversity-indices distribution as well as the assumption of normality, Figures A2 and A3 in S2 File) in order to eliminate effects of linear dependency attributable to the combination of three variables in one forest management intensity index as well as to eliminate perfect multicollinearity of the *exploratory* variable.

*A priori*, we fitted those environmental variables with an increase of more than 50% of the mean square error—in the case of omission—together with the marked highest IncNodePurity-values (derived from a random forest) in negative binomial-, gamma- or Gaussian-GLMMs in a sequence according to their importance (Tables 1–3, after [5]). This was followed by model dredging. The dredge function (implemented in the MuMIn package) was applied for model simplification [61] based on the highest Akaike weight. Model dredging retains model combinations with the most likely combinations of predictor variables [62,63].

**Table 1:** Statistical characteristics of models comparing the total abundance of silphids in diverse forest types.

	Silphidae			
Random effect variance (group = plot): 0.118, StdDev: 0.343				
Negative binomial dispersion parameter: 19.963 (Stderr: 11.898)				
	*F*	*Estimated slope*	*StdError of estimated slope*	*P*
*Abundance*				
Fine sand	3.384	-0.002	< 0.001	0.072
Mean ambient temperature	**10.659**	0.300	0.092	**0.002**
Random effect variance (group = plot): 0.092, StdDev: 0.303				
Negative binomial dispersion parameter: 4.036 (Stderr: 1.851)				
	*F*	*Estimated slope*	*StdError of estimated slope*	*P*
*Abundance*				
SMI (Silvicultural Management Intensity Index)	0.111	0.190	0.569	0.740
	***N*. *vespilloides***			
Random effect variance (group = plot): 0.199, StdDev: 0.446				
Negative binomial dispersion parameter: 9.890 (Stderr: 10.243)				
	*F*	*Estimated slope*	*StdError of estimated slope*	*P*
*Abundance*				
Fine sand	4.050	-0.002	0.001	0.050
Mean ambient temperature	**17.199**	0.410	0.099	**< 0.001**
Random effect variance (group = plot): 0.454, StdDev: 0.674				
Negative binomial dispersion parameter: 1.001 (Stderr: 0.002)				
	*F*	*Estimated slope*	*StdError of estimated slope*	*P*
*Abundance*				
SMI (Silvicultural Management Intensity Index)	0.148	0.249	0.646	0.702
	***N*. *investigator***			
Random effect variance (group = plot): < 0.001, StdDev: 0.002				
Negative binomial dispersion parameter: 7.266 (Stderr: 1.541)				
	*F*	*Estimated slope*	*StdError of estimated slope*	*P*
*Abundance*				
Bulk density	4.214	0.756	0.368	0.046
Mean ambient temperature	**42.181**	-0.569	0.088	**< 0.001**
Soil type	**13.006**			**< 0.001**
		Cambisol: -0.165	0.376	
		Leptosol: -0.250	0.428	
		Luvisol: -1.980	0.475	
		Stagnosol: -2.131	0.644	
Random effect variance (group = plot): 0.046, StdDev: 0.214				
Negative binomial dispersion parameter: 7.079 (Stderr: 2.319)				
	*F*	*Estimated slope*	*StdError of estimated slope*	*P*
*Abundance*				
Exploratory	**39.518**			**< 0.001**
		HEW: -1.990	0.296	
		SEW: -1.083	0.207	
SMI (Silvicultural Management Intensity Index)	0.627	0.420	0.531	0.432
	***N*. *humator***			
Random effect variance (group = plot): < 0.001, StdDev: 0.001				
Negative binomial dispersion parameter: 3.638 (Stderr: 1.582)				
	*F*	*Estimated slope*	*StdError of estimated slope*	*P*
*Abundance*				
Management system	**4.583**			**0.007**
		extensively managed: 1.649	1.079	
		selection system: 0.447	0.771	
		unmanaged: 1.271	0.382	
Mean ambient temperature	**14.731**	0.457	0.119	**< 0.001**
Soil type	2.032			0.107
		Cambisol: 0.012	0.487	
		Leptosol: -1.041	0.740	
		Luvisol: 0.176	0.569	
		Stagnosol: -1.307	0.740	
Random effect variance (group = plot): < 0.001, StdDev: 0.005				
Negative binomial dispersion parameter: 0.698 (Stderr: 0.168)				
	*F*	*Estimated slope*	*StdError of estimated slope*	*P*
*Abundance*				
SMI (Silvicultural Management Intensity Index)	**7.953**	-3.396	1.204	**0.007**

Results of negative binomial-GLMMs (plot as random effect) comparing the total abundance of all silphid beetle taxa and of the single silphid species *Nicrophorus vespilloides*, *N*. *investigator*, and *N*. *humator* in diverse forest types in three regions (AEW = Alb Experimental plot Wald (in English: forest), HEW = Hainich Experimental plot Wald (in English: forest), SEW = Schorfheide Experimental plot Wald (in English: forest)). Bold text indicates significant effects (α = 0.05). Important environmental variables (Figures A1—A4 in S1 File) were fitted first, according to their importance. For model dredging, model simplification based on Akaike information criterion AIC (dredge function implemented in the MuMIn package) was performed.

**Table 2:** Results of negative binomial-GLMMs comparing species richness of the taxon Silphidae in the different forest types in three regions.

	Silphidae			
Random effect variance (group = plot): < 0.001, StdDev: 0.002				
Negative binomial dispersion parameter: 1.001 (Stderr: < 0.001)				
	*Deviance*	*Estimated slope*	*StdError of estimated slope*	*P*
*Species richness*				
Model not significant	1.773			0.183
Clay		> -0.001	< 0.001	
Random effect variance (group = plot): < 0.001, StdDev: 0.002				
Negative binomial dispersion parameter: 1.001 (Stderr: < 0.001)				
	*F*	*Estimated slope*	*StdError of estimated slope*	*P*
*Species richness*				
SMI (Silvicultural Management Intensity Index)	0.008	0.043	0.473	0.928

Plot was used as a random effect. Important environmental variables (Fig A5 in S1 File) were fitted first, according to their importance. For model dredging, model simplification based on Akaike information criterion AIC (dredge function implemented in the MuMIn package) was performed.

**Table 3:** Results of models comparing Shannon’s diversity and Simpson’s dominance of silphids in diverse forest types.

	Silphidae			
	Gaussian-GLMM			
Random effect variance (group = plot): < 0.001, StdDev: 0.001				
Residual variance: 0.211 (Stderr: 0.021)				
	*F*	*Estimated slope*	*StdError of estimated slope*	*P*
*Shannon’s diversity*				
Mean ambient temperature	**7.840**	-0.126	0.045	**0.008**
Soil type	**20.541**			**< 0.001**
		Cambisol: -0.519	0.155	
		Leptosol: -0.526	0.187	
		Luvisol: -0.907	0.204	
		Stagnosol: -0.807	0.248	
	Gaussian-GLMM			
Random effect variance (group = plot): < 0.001, StdDev: 0.001				
Residual variance: 0.229 (Stderr: 0.022)				
	*F*	*Estimated slope*	*StdError of estimated slope*	*P*
*Shannon’s diversity*				
Exploratory	**7.606**			**0.001**
		HEW: -0.439	0.139	
		SEW: -0.218	0.108	
SMI (Silvicultural Management Intensity Index)	1.284	-0.368	0.325	0.263
				
	gamma-GLMM			
Random effect variance (group = plot): 0.027, StdDev: 0.163				
Gamma shape parameter: 403.43 (Stderr: 0.043)				
	*F*	*Estimated slope*	*StdError of estimated slope*	*P*
*Simpson’s dominance*				
Fine silt	4.037	-0.002	0.001	0.051
Mean ambient temperature	**23.634**	-0.184	0.038	**< 0.001**
Soil type	**15.703**			**< 0.001**
		Cambisol: -0.360	0.106	
		Leptosol: -0.365	0.126	
		Luvisol: -0.658	0.122	
		Stagnosol: -0.519	0.139	
	gamma-GLMM			
Random effect variance (group = plot): 0.036, StdDev: 0.191				
Gamma shape parameter: 403.43 (Stderr: 0.035)				
	*F*	*Estimated slope*	*StdError of estimated slope*	*P*
*Simpson’s dominance*				
Exploratory	**22.869**			**< 0.001**
		HEW: -0.360	0.069	
		SEW: -0.278	0.065	
SMI (Silvicultural Management Intensity Index)	2.300	-0.291	0.192	0.136

Statistical characteristics are shown for all three regions. For Gaussian- and gamma-GLMMs, the link = “log”. Bold text indicates significant effects (α = 0.05). Important environmental variables (Figures A6 and A7 in S1 File) were fitted first, according to their importance. For model dredging, model simplification based on Akaike information criterion AIC (dredge function implemented in the MuMIn package) was performed.

Finally, we calculated Shannon’s diversity as ‘–Σ*P*_i_ * ln(*P*_i_)’ where *P*_i_ is the proportion of individuals belonging to species *i*, and Simpson’s dominance as ‘1/Σ*P*_i_^2^’ (formulae from [64]; [65]). Morris et al. (2014) suggest that the inclusion of multiple diversity measures, spread along Hill’s continuum [66], provides researchers with a more complete understanding of the way that shifts in abundant and rare species drive interactions. Following their recommendation, we included not only species richness (sensitive to rare species, [67]), but also, as aforementioned, Shannon’s diversity (equally sensitive to abundant and rare species; [67]) and Simpson’s dominance (sensitive to abundant species, more common than Simpson’s diversity; [68]). The random forest approach and model dredging for the quantification of the relative importance of environmental variables on silphid beetle diversity were calculated as described in detail above (for plots of variable distribution and for detecting departures from normality, see Figures A2 and A3 in S2 File).

## Results

**Fig 1:**
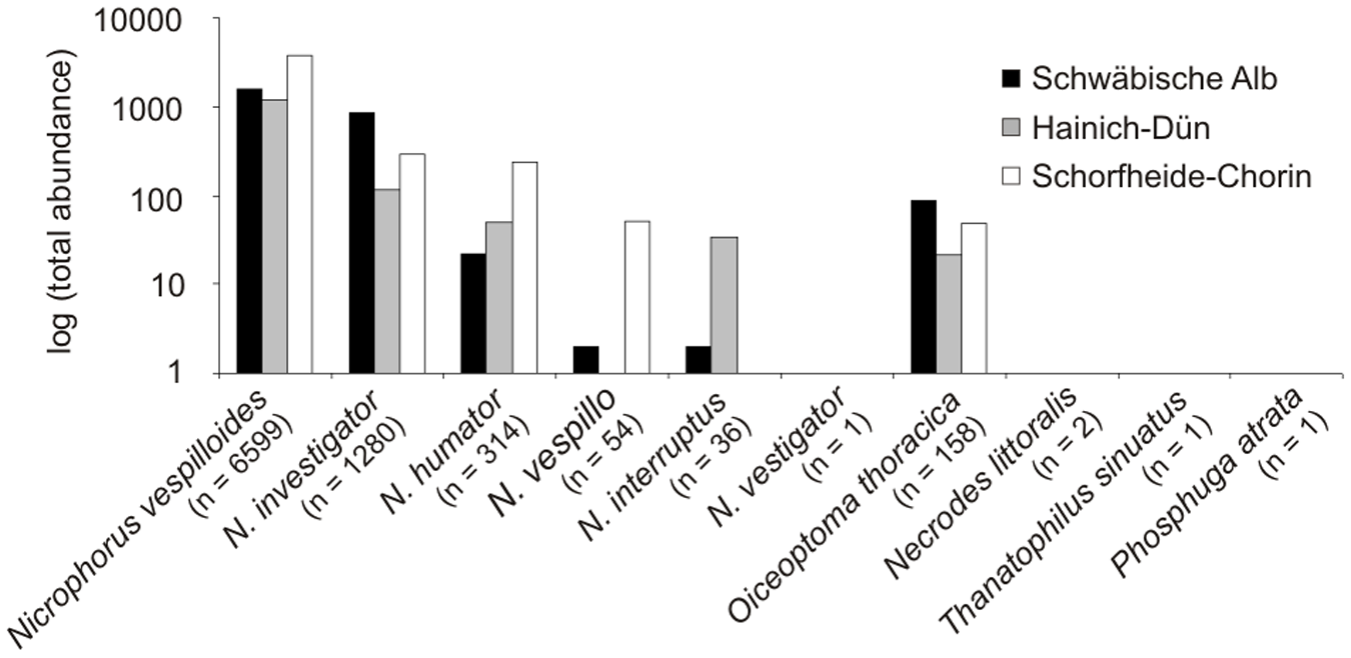
Total abundance of individuals of each trapped silphid beetle species. Y-axis is shown in logarithmic scale. Abundance data are separated for each exploratory. Exposure of 61 piglet cadavers from August 4^th^ until September 4^th^ 2014 (*N*. = *Nicrophorus*).

During the whole fieldwork period, we trapped 8446 silphid beetle individuals of 10 species on the periphery of 61 exposed piglet cadavers: *Nicrophorus vespilloides* (n = 6599), *N*. *investigator* (n = 1280), *N*. *humator* (n = 314), *Oiceoptoma thoracica* (n = 158), *N*. *vespillo* (n = 54), *N*. *interruptus* (n = 36), *Necrodes littoralis* (n = 2), *Thanatophilus sinuatus* (n = 1), *N*. *vestigator* (n = 1), and *Phosphuga atrata* (n = 1) (Fig 1). In the respective controls, we trapped only one individual of the species *N*. *vespilloides*. The number of individuals trapped per plot ranged from 13 (one single plot in Hainich-Dün) to 409 individuals (one single plot in Schorfheide-Chorin). Cadaver-baited traps captured significantly more silphid beetles compared with unbaited control traps across all three regions (Kruskal-Wallis test, Chi^2^ = 103.01, df = 1, P < 0.001). Species number per plot ranged from one captured silphid species in Hainich-Dün to seven captured species in Schorfheide-Chorin.

### Effects of environmental characteristics on overall silphid beetle abundance

**Fig 2:**
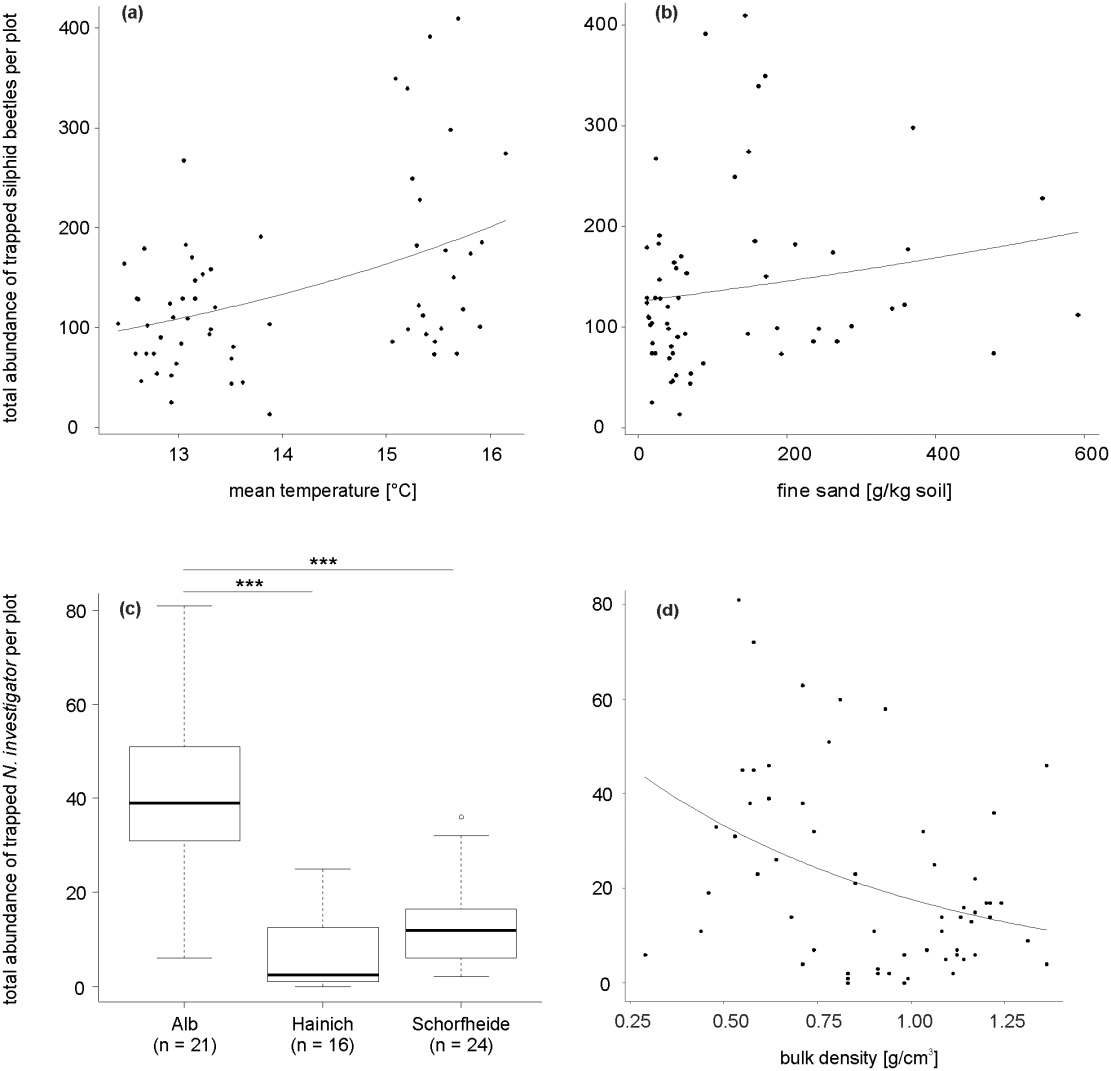
Effects of environmental characteristics on overall silphid beetle and *N*. *investigator* abundance. Relationship between total abundance of trapped silphid beetles per plot and **a** mean ambient temperature and **b** fine sand content. Relationship between total abundance of trapped *N*. *investigator* per plot and **c** region and **d** bulk density. **a** & **b**: observed values (circles) and predicted values (connected by a line) for the negative binomial-GLMM model (deviance = 10.39, P = 0.006), **c**: box plot showing the median, the 75% percentile, the 25% percentile, the highest non-extreme value, the smallest non-extreme value, and the extreme values inside a category (negative-binomial GLMM model comparison, deviance = 49.19, P < 0.001; Tukey tests, ***P < 0.001), **d**: observed values (circles) and predicted values (connected by a line) for the negative-binomial GLMM model (deviance = 56.44, P < 0.001).

Two abiotic environmental variables influenced the abundance of members of all captured silphid beetle taxa (Table 1). From overall two predictor variables in the simplified model, with a random effect variance of 0.18 (negative binomial-GLMM, deviance = 10.39, P = 0.006), ‘mean ambient temperature’ significantly affected the abundance of Silphidae. The same was tendentially true for the variable ‘fine sand’ (Table 1). Across all three regions, total silphid beetle abundance increased with higher mean ambient temperatures (Fig 2a). Overall beetle abundance tended to increase with an increasing fine-sand content (Fig 2b). Forest management intensity had no significant effect on overall silphid beetle abundance (Table 1).

### Effects of environmental characteristics on the abundance of *N*. *vespilloides*

**Fig 3:**
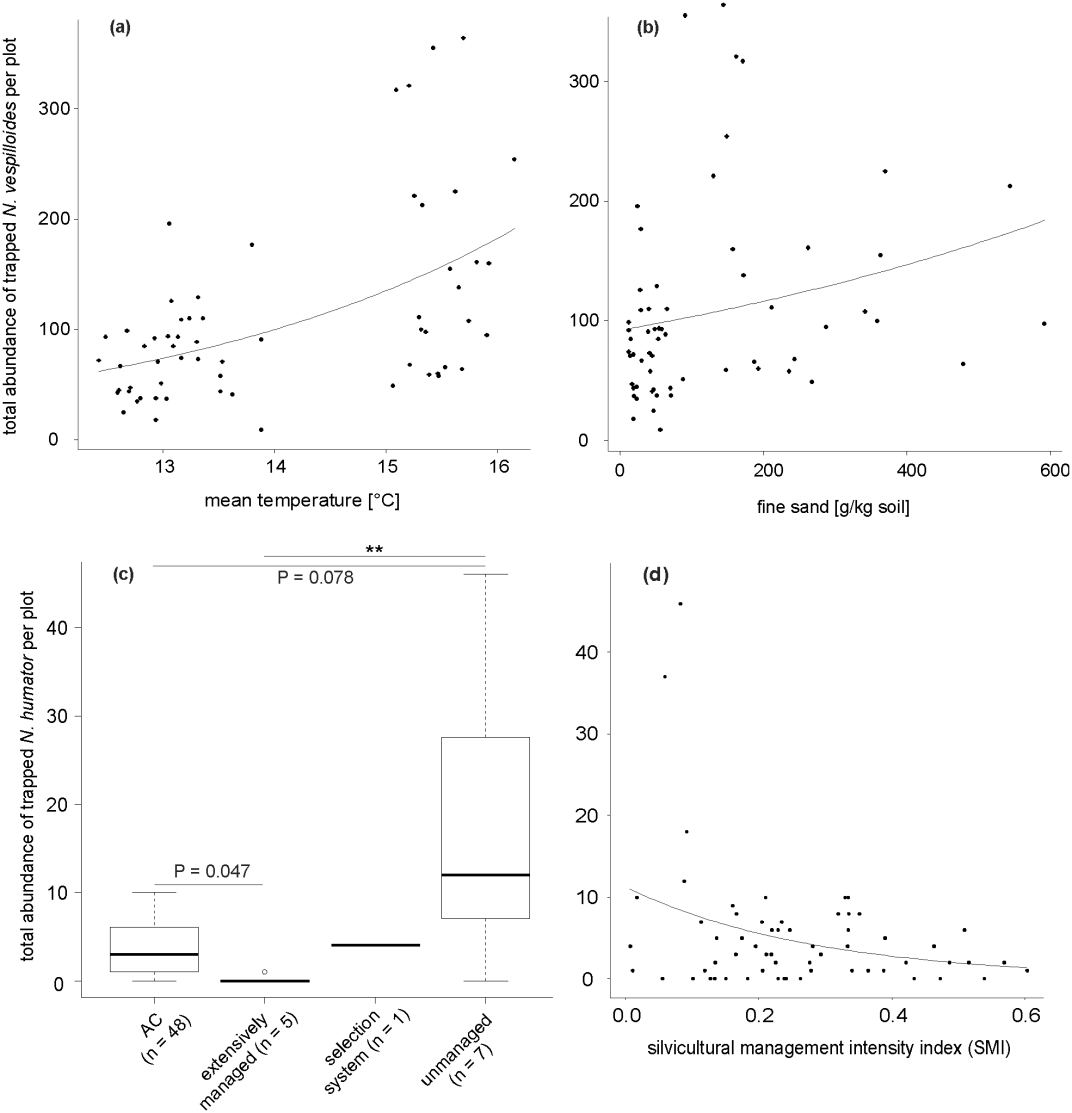
Effects of environmental characteristics on *N*. *vespilloides* and *N*. *humator* abundance. Relationship between total abundance of trapped *N*. *vespilloides* per plot and **a** mean ambient temperature and **b** fine sand content, and between total abundance of trapped *N*. *humator* per plot and **c** management system (AC = age class, n = number of plots per form of forest management) and **d** silvicultural management intensity (SMI). **a** & **b**: observed values (circles) and predicted values (connected by a line) for the negative binomial-GLMM model (deviance = 15.25, P < 0.001). **c**: box plot showing the median, the 75% percentile, the 25% percentile, the highest non-extreme value, the smallest non-extreme value, and the extreme values inside a category (deviance = 55.73, P < 0.001; Tukey tests, **P < 0.01). **d**: observed values (circles) and predicted values (connected by a line) for the negative binomial-GLMM model (deviance = 5.04, P = 0.025).

Two abiotic environmental variables influenced the abundance of *N*. *vespilloides* individuals (Table 1). From overall two predictor variables in the simplified model, with a random effect variance of 0.20 (negative binomial-GLMM, deviance = 15.25, P < 0.001), ‘mean ambient temperature’ significantly influenced the abundance of *N*. *vespilloides* individuals (Table 1). Across all three regions, the total abundance of *N*. *vespilloides* increased with higher ambient temperatures (Fig 3a). The same was tendentially true for higher fine-sand contents (Fig 3b, Table 1). Silvicultural management intensity (expressed as an index) did not affect *N*. *vespilloides* abundance (Table 1).

### Effects of environmental characteristics on the abundance of *N*. *investigator*

Total abundance of *N*. *investigator* was higher in the Schwäbische Alb when compared with Hainich-Dün and Schorfheide-Chorin, respectively (negative binomial-GLMM, deviance = 49.19, P < 0.001, Fig 2c, Table 1 (variable *Exploratory*)). From, in total, three predictor variables in the simplified model, with a random effect variance of less than 0.001 (negative binomial-GLMM, deviance = 56.44, P < 0.001), the two environmental variables ‘mean ambient temperature’ and ‘soil type’ significantly influenced the abundance of *N*. *investigator* individuals (Table 1). Bulk density tendentially influenced beetle abundance (Table 1). Across all three regions, the total abundance of *N*. *investigator* decreased with higher mean ambient temperatures (Fig A4a in S2 File). Concerning soil type, the abundance of *N*. *investigator* was significantly higher on Leptosol soils compared with Luvisol and Stagnosol soil types, respectively (Fig A4b in S2 File). Furthermore, total abundance of *N*. *investigator* tended to decrease with higher bulk densities (Fig 2d). Silvicultural management intensity (expressed as an index) had no effect on *N*. *investigator* abundance (Table 1).

### Effects of environmental characteristics on the abundance of *N*. *humator*

From, in total, three predictor variables in the simplified model, with a random effect variance of less than 0.001 (negative binomial-GLMM, deviance = 55.73, P < 0.001), ‘management system’ and ‘mean ambient temperature’ significantly influenced the abundance of *N*. *humator* individuals (Table 1). Across all three regions, the total abundance of *N*. *humator* was higher in unmanaged forests compared with those that were extensively managed. The latter forest type also showed a tendentially lower abundance of *N*. *humator* when compared with age-class forests (Fig 3c). In addition, tendentially more *N*. *humator* individuals were captured in unmanaged forests compared with age-class forests (Fig 3c). Across all three regions, the total abundance of *N*. *humator* increased with higher mean ambient temperatures (Fig A5 in S2 File). Silvicultural management intensity (expressed as an index) had an effect on *N*. *humator* abundance (negative binomial-GLMM, deviance = 5.04, P = 0.025). Across all three regions, higher silvicultural management intensity resulted in a decrease of abundance of *N*. *humator* (Fig 3d, Table 1).

### Effects of environmental characteristics on species richness

The simplified model, with a random effect variance of less than 0.001, and with the abiotic environmental variable ‘clay’ as the only predictor variable (negative binomial-GLMM, deviance = 1.77, P = 0.183) was not significant (Table 2). Forest management intensity had no effect on silphid species richness (negative binomial-GLMM, deviance = 0.01, P = 0.927, Table 2).

### Effects of environmental characteristics on the diversity (Shannon’s diversity & Simpson’s dominance) of the taxon Silphidae

**Fig 4:**
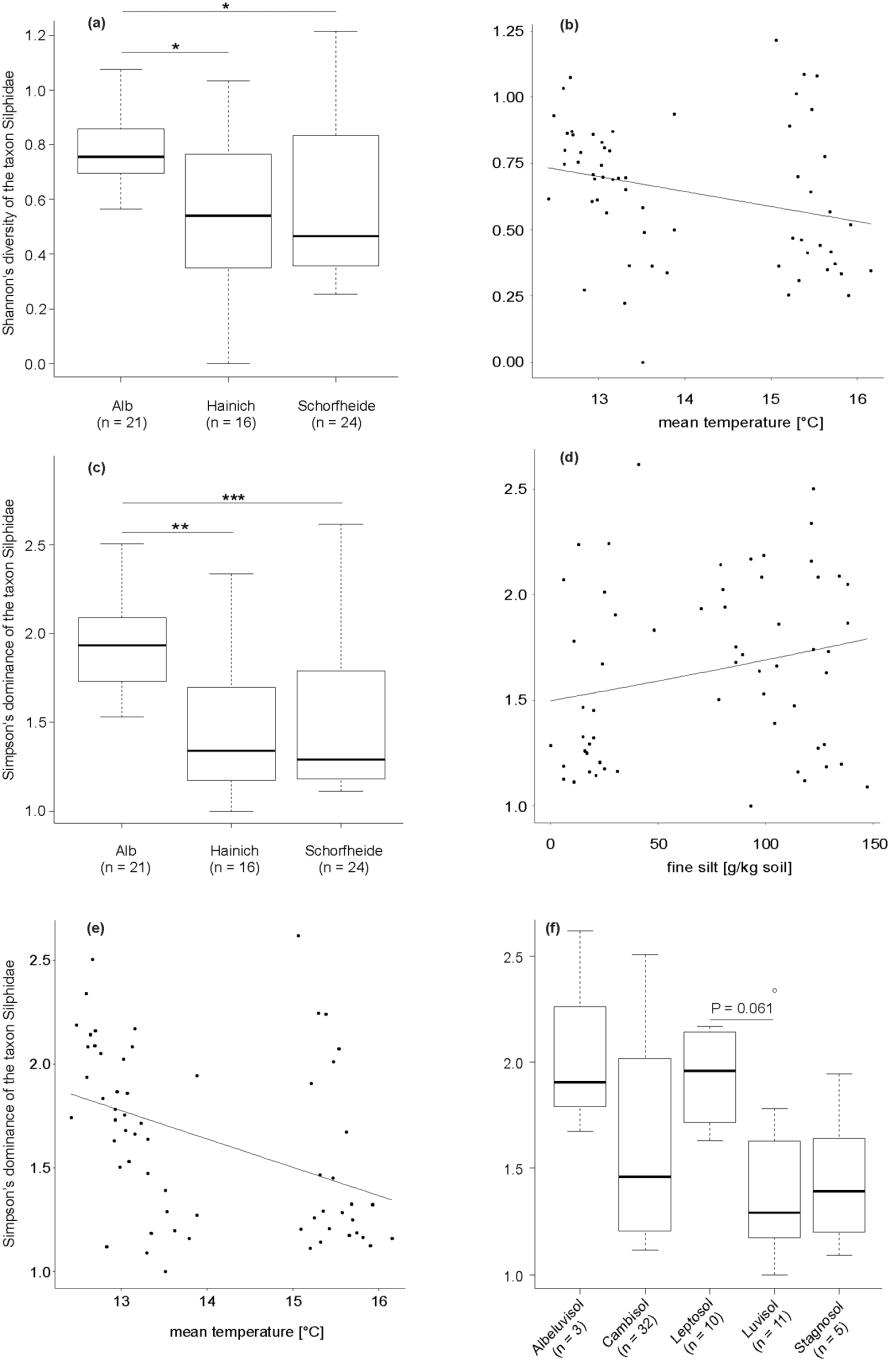
Effects of environmental characteristics on the diversity of the taxon Silphidae. Relationship between Shannon’s diversity of the taxon Silphidae and **a** region and **b** mean ambient temperature. Relationship between Simpson’s dominance of the taxon Silphidae and **c** region, **d** fine silt content, **e** mean ambient temperature, and **f** soil type (n = number of plots per soil type). Observed values (circles) and predicted values (connected by a line) for the Gaussian-GLMM model (**b**: deviance = 19.66, P = 0.001) and for gamma-GLMM models (**d** & **e**: deviance = 40.57, P < 0.001). **a**: box plot showing the median, the 75% percentile, the 25% percentile, the highest non-extreme value and the smallest non-extreme value inside a category (Gaussian-GLMM, deviance = 11.11, P = 0.011; Tukey tests, *P < 0.05), **c**: box plot showing the median, the 75% percentile, the 25% percentile, the highest non-extreme value and the smallest non-extreme value inside a category (gamma-GLMM, deviance = 25.15, P < 0.001; Tukey tests, **P < 0.01, ***P < 0.001), and **f**: box plot showing the median, the 75% percentile, the 25% percentile, the highest non-extreme value, the smallest non-extreme value, and the extreme values inside a category (gamma-GLMM, deviance = 40.57, P < 0.001; Tukey tests, P > 0.05).

Several abiotic environmental variables influenced the diversity of all captured silphid beetle individuals (Table 3). From, in total, two predictor variables in the simplified model, with a random effect variance of less than 0.001 (Gaussian-GLMM, deviance = 19.66, P = 0.001), ‘mean ambient temperature’ and ‘soil type’ significantly influenced Shannon’s diversity of the taxon Silphidae (Table 3). Across all three regions, Shannon’s diversity of the taxon Silphidae decreased with higher mean ambient temperatures (Fig 4b, Table 3). Concerning ‘soil type’, Tukey posthoc tests revealed no significant differences (as well as no tendencies) between different soil types regarding Shannon’s diversity (Gaussian-GLMM, deviance = 19.66, P = 0.001; Tukey tests, P > 0.05). Regionally, Shannon’s diversity was higher in the Schwäbische Alb region compared with Hainich-Dün and Schorfheide-Chorin, respectively (Gaussian-GLMM, deviance = 11.11, P = 0.011; Tukey tests, *P < 0.05, Fig 4a, Table 3 (variable *Exploratory*)). Silvicultural management intensity (expressed as an index) had no effect on Shannon’s diversity in all captured silphid beetles (Table 3). From, in total, three predictor variables in the simplified model, with a random effect variance of 0.027 (gamma-GLMM, deviance = 40.57, P < 0.001), ‘mean ambient temperature’ and ‘soil type’ significantly influenced Simpson’s dominance of the taxon Silphidae (Table 3). Across all three regions, Simpson’s dominance declined with higher mean ambient temperatures (Fig 4e). Simpson’s dominance of all captured silphid beetles also tendentially declined on Luvisol soils when compared with Leptosol soils (Fig 4f, Tukey tests, P > 0.05, Luvisol ~ Leptosol: P = 0.061). Across all three regions, Simpson’s dominance of the taxon Silphidae tended to increase with higher fine silt contents (Fig 4d, Table 3). Regionally, Simpson’s dominance was higher in the Schwäbische Alb region compared with Hainich-Dün and Schorfheide-Chorin, respectively (gamma-GLMM, deviance = 25.15, P < 0.001; Tukey tests, **P < 0.01, ***P < 0.001, Fig 4c, Table 3 (variable *Exploratory*)). Forest management intensity had no effect on Simpson’s dominance in all captured silphid beetles (Table 3).

## Discussion

During our large scale carrion experiment we collected data from silphid beetles trapped on 61 piglet cadavers decomposing in differently managed forest types in three regions of Germany. In addition to ambient temperature, several habitat parameters, especially soil characteristics, had a strong impact on the carrion beetle abundance and diversity.

### General results

During our field experiment, we trapped and identified 10 species of the taxon Silphidae on the periphery of exposed piglet cadavers. The most frequent species was the burying beetle *Nicrophorus vespilloides*, followed by *N*. *investigator* and *N*. *humator*. A similar result was reported on five deer carcasses in the Sonian Forest in Belgium. Haelewaters et al. (2015) recorded a total of 9 species of carrion beetles (the most diverse taxon in their study) with *N*. *vespilloides* as the most abundant, followed by *Necrodes littoralis* and *N*. *humator* [69].

Abundance and species richness of silphids varied considerably between trapping sites from only 13 individuals in the Hainich-Dün region and up to 409 individuals in the Schorfheide-Chorin region. This is in agreement with the high variation in registered species numbers, rising from one on a specific plot in Hainich-Dün, two on a specific plot in the Schwäbische Alb, and up to seven on a specific plot in Schorfheide-Chorin. Lange et al. (2014) reported similar results in their study with ground-dwelling carabid and staphylinid beetles within the Biodiversity Exploratories. In both beetle taxa, they found most species in the Schorfheide-Chorin region followed by Hainich-Dün and the Schwäbische Alb. During our fieldwork period, we trapped 52 out of 54 (96.3%) individuals of the open-habitat species *Nicrophorus vespillo* [24,70] in the Schorfheide-Chorin region only. Out of these 52 individuals, 23 individuals (44%) were retrieved from cadavers exposed in open pine tree forests that are typical for this region. Appropriately, Barton et al. (2017) argued that cadaver associated insect communities vary between locations, and that, consequently, detailed case studies are necessary for identifying similarities and differences among contrasting habitats. They found significant effects of habitat type (contrasting grassland and tree habitats) and time, but not their interaction, on the composition of the entire carrion insect community [71]. For example, the fly species *Chrysomya varipes* (Diptera: Calliphoridae) was more abundant under trees than in grassland during active decay of rabbit carcasses exposed in southeastern Australia [71]. Because habitat type (open or closed, as expressed in the variable “crown closure”) was not a significant factor in our forest study sites (see Figures A1—A7 in S1 File), we highly encourage a comparative future study in open grasslands of the Biodiversity Exploratories, as proposed by Barton et al. (2017). We also speculate that the abundance of mammals, in their role as potential cadaver suppliers, could be another important factor. Holloway and Schnell (1997) showed that American burying beetles preferred sites in which small mammals were relatively abundant, irrespective of the predominant habitat [72]. The unequally distributed numbers of silphid beetle individuals and species across the three studied regions, as we have found in this study, might be associated with the abundance of wild animals, in particular red deer (*Cervus elaphus*), in German forests. Despite the red deer not being a small mammal, it can be found exclusively in the region of Schorfheide-Chorin with a high abundance of 40 to 70 individuals per 1000 hectares [73] for a long period of time [74]. The higher availability of large cadavers in forests of the Schorfheide-Chorin region might have been the precondition for the development of higher population densities of necrophagous silphid beetles compared with those in the red-deer-free zones in Hainich-Dün and the Schwäbische Alb. This is probably especially true for Silphinae individuals. They not only feed, but also reproduce on large cadavers (much larger than our exposed piglets; [24,45,49,75]). However, on our piglet cadavers (1.44 kg average weight) the collected Silphinae species with the highest frequency was *Necrodes littoralis*, with only two individuals. We explain this lack of diversity in the Silphinae group by the important factor carcass mass. The mass of a cadaver is especially important for cadaver odor emission during decomposition, which influences the pattern, rate and duration of cadaver decomposition [76], as well as carrion entomofauna [77]. The findings of Matuszewski et al. (2015) indicate that even cadavers of 23 kg in weight—a standard in forensic decomposition studies—give an incomplete picture of the carrion entomofauna. In their study with four levels of carrion mass, they detected Silphinae individuals on small carcasses (5–15 kg) up to large carcasses (55–70 kg). Therefore, to determine more exactly the diversity of the Silphinae group, further experiments with cadavers of at least 5 kg (still better 55 kg) are needed. Another important aspect that potentially influences carrion entomofauna is time of the year. Since *N*. *littoralis* and *T*. *sinuatus* are active from April till September [24], their near absence in our pitfall traps could be explained by our fieldwork period in late summer (beginning of August till beginning of September). But one has to keep in mind that *Nicrophorus* sp., despite feeding on large cadavers, is much more dependent on small carrion [35], which they require for reproduction. Therefore, the abundance of small carrion in the three distinct regions of the Biodiversity Exploratories has to be examined in future investigations.

### Effects of land use on silphid beetle abundance and diversity

Land use intensity and consequently changes in certain environmental characteristics of a given habitat may influence arthropod communities directly by reducing their population size (during harvest activities) or indirectly by affecting habitat heterogeneity, habitat availability, or prey (and consequently carcass) availability [5]. For measuring land use intensity, one of the main forest management activities—wood harvest—was used in several studies [78–81]. Thereby, several measures of logging intensity have been recommended, like the harvested basal area, stem number, volume, or the respective attributes of residual trees [82]. Despite that it has been demonstrated that harvest intensity has a strong impact on the diversity of organisms [78,80,81], harvesting is just one important component of forest management intensity. Gossner et al. (2014) compared different approaches of quantifying the intensity of land use for their ability to explain differences in diversity and community composition between forest stands. They found that a quantitative measure for land use intensity, like the silvicultural management intensity indicator (SMI), can help to understand even more subtle relationships between human disturbance and the biodiversity of organisms [82]. SMI, the preferred management intensity indicator we used in our study, turned out to be a useful tool for the quantification of land use intensity in forests, even in different forest ecosystems worldwide [82]. This index was developed to quantify silvicultural land use intensity based on the two most influential management decisions on the strategic and the operational level. The first is related to tree species selection and stand age (rotation period) and the second reflects site productivity, the control of stand density by thinnings and harvests and consequently biomass removal relative to the carrying capacity [46, 82]. All these characteristics were combined into the density and risk components of the SMI-index [46].

In our study, *N*. *humator* was the only silphid species that was trapped on piglet cadavers that occurred in lower abundance in locations with higher forest utilization (expressed by a higher SMI-index). This might lead to the designation of *N*. *humator* as a valuable indicator species for forest managers when assessing anthropogenic disturbances in German forests. Our finding is supported by the unexpectedly low numbers of *N*. *humator* in a field trapping study in Poland that used small and large rodent cadavers as bait [83]; the authors suggested habitat fragmentation as a possible cause for their results. Trumbo and Bloch (2000) showed that fragmented habitats result in decreasing populations of large burying beetles such as the endangered American species *N*. *americanus* or *N*. *humator* (one of the largest burying beetle species) [84]. Similar results concerning habitat fragmentation have been shown for beetles of the taxon Carabidae [85]. Our finding, showing that *N*. *humator* is more abundant in unmanaged stands compared with age-class forests and with extensively managed stands, underpins its susceptibility to anthropogenic altered habitats. Furthermore, the same result reveals that age-class forest management has a higher conservation value for the species *N*. *humator* compared with the management strategy of extensive forest use.

### Effects of environmental characteristics on silphid beetle abundance and diversity

In our study, we found that factors of the environment influence the abundance of silphid beetles and their diversity (Shannon’s diversity and Simpson’s dominance). Effects of environmental characteristics on species richness could not be detected. We have shown that higher ambient temperatures and higher fine sand content in soil profiles have a positive effect not only on the abundance of the overall silphid beetle taxon, but also for single silphid species, e.g., *N*. *vespilloides* or *N*. *humator*. As has long been known, soil parameters are an important factor for silphid species [25,36,53]. On the one hand, this might be because they pupate underground [25]; on the other hand, the entire reproductive bout of *Nicrophorus* spp. (98.1% of our trapped specimens) takes place below ground after they bury small vertebrate cadavers in the soil as food for their offspring [35,36]. Therefore, Pukowski (1933) and Novák (1961 [86] and 1962 [87]) have proposed that some soils are more suitable for maintaining a stable environment in terms of moisture and temperature, which is beneficial for the Silphidae taxon. Laboratory experiments have revealed that burying beetles are able to distinguish among different soil types and choose the best substrate for digging [88]. In agreement with this, we have found a higher abundance of the single silphid species *N*. *investigator* on loose soils (particularly suitable for digging) with lower bulk densities (Fig 2d). Possible explanations of our findings are, first, that the digging process is less energetically costly in loose soils and, second, that looser soils are favored by burying beetles because of a more rapid and deeper burying process and therefore less inter- and intraspecific competition. Our results are also in accordance with the finding that the trapping success of the American burying beetle *N*. *americanus* increases as the percentage of sand increases and the percentage of clay and silt decreases [89,90].

Our data reveal clear effects of soil type, soil texture, and ambient temperature on Shannon’s diversity and Simpson’s dominance in ten trapped carrion beetle species. For example, Simpson’s dominance of silphids as well as the abundance of *N*. *investigator* is, on average, highest on Leptosol soils (Fig A4b in S2 File, Fig 4f). This soil type is widespread in the Schwäbische Alb region [47], which shows the highest Shannon’s diversity as well as Simpson’s dominance of the taxon Silphidae (Fig 4a and 4c), and furthermore, the highest abundance of *N*. *investigator* as well (Fig 2c). Across all three regions, another soil type, the Albeluvisol, showed the highest measured Simpson’s dominance of the Silphidae taxon (Fig 4f). Albeluvisol soils are most widespread on the Mittelbrandenburg Plate [91] and show, in beech-dominated forests of the Biodiversity Exploratories, high fine silt contents. This corresponds to our finding of a higher Simpson’s dominance in the silphid beetle group on soils with higher fine silt contents (Fig 4d).

### Influence of climatic conditions on silphid beetle diversity

All levels of biodiversity, from organisms to biomes, are anticipated to be affected by the multiple components of climate change [92,93]. The functioning and resilience of ecosystems might be affected by the possible directional selection and rapid migration (decrease of genetic diversity of populations) caused by climate change [94,95]. We have found that higher ambient temperatures clearly reduce Shannon’s diversity and Simpson’s dominance (Fig 4b and 4e). In a decomposition study involving traps baited with small mammal carrion (two *Mus musculus* cadavers, body mass approximately 20 g for each), Farwig et al. (2014) reported that lower decomposition rates at lower temperatures seem to be caused by the absence of large silphid beetles such as *N*. *vespilloides*, *N*. *investigator*, and *O*. *thoracica*. Therefore, they conclude that global environmental change will presumably affect the decomposition of cadavers and consequently nutrient recycling in ecosystems through the reorganization of the composition of carcass-scavenging assemblages [63]. Small carrion was used in the study of Farwig et al. (2014), and hence, they encouraged the performance of further studies with large cadavers, like ours, for the generalization of their conclusions. Our result, based on Shannon’s diversity and Simpson’s dominance, indicates as well that global environmental change (in more detail, global warming) might affect the overall species composition of the necrophilous and/or necrophagous insect community on large vertebrate cadavers. This is also demonstrated by the lowest abundance of *N*. *investigator* when ambient temperatures are highest (Fig 2a). Such reductions of silphid beetle diversity and abundance, the latter at least for particular species, might alter the predictable pattern of the succession of cadaver-associated insects [96] and the whole decomposition process and consequently nutrient recycling in ecosystems [63]. However, one has to keep in mind that differential responses of scavenging arthropods and vertebrates to forest loss might maintain ecosystem function in a heterogeneous landscape [97]. Sugiura et al. (2013) demonstrated the functional redundancy of burying beetles as well as the maintenance of carrion removal by the differential responses of burying beetles and scavenging vertebrates. DeVault et al. (2011) found that these two effects could be mediated by the scavenging community composition in a fragmented, agricultural landscape [10]. Facultative scavenging mammalian midtrophic level predators (so called mesopredators) dominated carrion acquisition over invertebrate and microbial competitors [10] likely due to the elevated abundance of mesopredators in their study landscape. This suggests, that the predominance of generalists, such as vertebrate scavengers, over insect specialists such as carrion beetles (at least in regions where carrion beetles are uncommon), may have wider implications. In general, vertebrate scavenging represents the widest dispersal of nutrients and energy from cadavers as movement of vertebrates scale away to the broader landscape [14,98]. Therefore, the interesting question, how an altered carrion-consuming community impacts overall carrion ecology, needs further work in future.

## Conclusions

Our carrion ecology study examining 61 exposed *Sus scrofa domestica* cadavers across three regions in Germany suggests land use, forest soil characteristics, and overall climate as important factors determining the diversity of the cadaver-associated silphid beetle group. Our results designate loose soils with higher fine sand contents and lower ambient temperatures as suitable for undisturbed silphid beetle activity, the latter representing an important factor for rapid cadaveric nutrient recycling in intact ecosystems. Furthermore, the easy catchable single silphid species *Nicrophorus humator* appears to be an indicator species for human-induced forest disturbances. This implies that the two most influential management decisions on the strategic (tree species selection and rotation period) and the operational level (control of stand density by thinnings and harvests and consequently disturbance due to biomass removal), both expressed in the silvicultural management intensity indicator [46,82], have to be taken into account in addition to diverse environmental characteristics to protect a large carrion beetle species.

As a future perspective, we recommend an investigation of the effect of forest management on the overall decomposition rate of large exposed cadavers and on the succession of the primary insect taxa associated with carrion. We are presently examining this aspect inside of the framework of the Biodiversity Exploratories.

## Supporting information

S1 File (DOC)

S2 File (DOC)
