# Supplementary material for: Effects of abiotic environmental factors and land use on the diversity of carrion-visiting silphid beetles (Coleoptera: Silphidae): A large scale carrion study
Source: PLoS One. 2018 May 30;13(5):e0196839. doi: 10.1371/journal.pone.0196839 (PMC5976144; doi:10.1371/journal.pone.0196839)
Supplement: S1 File — (DOC) [file pone.0196839.s001.doc]

**Fig A1. Variable selection based on a random forest for prediction of silphid abundance.** The random forest method was used in order to determine those habitat variables that were more important for predicting the total abundance of all silphid beetle taxa. This was performed by running regression trees in a high number (here, number of runs = 10^5^, cf. [1]). This method [2,3] will work even for highly correlated variables, allowing similar important scores for each. *A priori*, for a negative binomial-GLMM (random effect at the plot level), we chose those environmental variables with an increase of more than 50% of the mean square error (%IncMSE) – in the case of omission – (Fine_Sand_.g.kg_soil., soil_moisture_10cm_..., Fine_Silt_.g.kg_soil. and mean_temperature) together with the marked highest IncNodePurity-value (soil_temperature_10cm) for a subsequent model simplification based on Akaike information criterion (AIC; dredge function implemented in the MuMIn package).

**Fig A2. Variable selection based on a random forest for prediction of *Nicrophorus vespilloides* abundance.** The random forest method was used in order to determine those habitat variables that were more important for predicting the total abundance of *N. vespilloides*. This was performed by running regression trees in a high number (here, number of runs = 10^5^, cf. [1]). This method [2,3] will work even for highly correlated variables, allowing similar important scores for each. *A priori*, for a negative binomial-GLMM (random effect at the plot level), we chose those environmental variables with an increase of more than 50% of the mean square error (%IncMSE) – in the case of omission – (mean_temperature, Fine_Sand_.g.kg_soil., soil_moisture_10cm_..., Fine_Silt_.g.kg_soil. and mineral_soil_pH) together with the marked highest IncNodePurity-value (soil_temperature_10cm) for a subsequent model simplification based on Akaike information criterion (AIC; dredge function implemented in the MuMIn package).

**Fig A3. Variable selection based on a random forest for prediction of *Nicrophorus investigator* abundance.** The random forest method was used in order to determine those habitat variables that were more important for predicting the total abundance of *N. investigator*. This was performed by running regression trees in a high number (here, number of runs = 10^5^, cf. [1]). This method [2,3] will work even for highly correlated variables, allowing similar important scores for each. *A priori*, for a negative binomial-GLMM (random effect at the plot level), we chose those environmental variables with an increase of more than 50% of the mean square error (%IncMSE) – in the case of omission – (mean_temperature, Fine_Sand_.g.kg_soil., soil_type, Fine_Silt_.g.kg_soil. and bulk_density_.g.cm3.) together with the marked highest IncNodePurity-value (exploratory) for a subsequent model simplification based on Akaike information criterion (AIC; dredge function implemented in the MuMIn package).

**Fig A4. Variable selection based on a random forest for prediction of *Nicrophorus humator* abundance.** The random forest method was used in order to determine those habitat variables that were more important for predicting the total abundance of *N. humator*. This was performed by running regression trees in a high number (here, number of runs = 10^5^, cf. [1]). This method [2,3] will work even for highly correlated variables, allowing similar important scores for each. *A priori*, for a negative binomial-GLMM (random effect at the plot level), we chose those environmental variables with an increase of more than 50% of the mean square error (%IncMSE) – in the case of omission – (soil_moisture_10cm_..., soil_type, Fine_Silt_.g.kg_soil., mineral_soil_pH, mean_temperature and Fine_Sand_.g.kg_soil.) together with the marked highest IncNodePurity-values (management_system and Clay_.g.kg_soil.) for a subsequent model simplification based on Akaike information criterion (AIC; dredge function implemented in the MuMIn package).

**Fig A5. Variable selection based on a random forest for prediction of silphid species richness.** The random forest method was used in order to determine those habitat variables that were more important for predicting the species richness of the taxon Silphidae limited by trapped beetle taxa *Nicrophorus vespilloides*, *N. investigator*, *N. humator*, *N. interruptus*, *N. vespillo*, *N. vestigator*, *Oiceoptoma thoracica*, *Necrodes littoralis*, *Thanatophilus sinuatus*, and *Phosphuga atrata*. This was performed by running regression trees in a high number (here, number of runs = 10^5^, cf. [1]). This method [2,3] will work even for highly correlated variables, allowing similar important scores for each. *A priori*, for a negative binomial-GLMM (random effect at the plot level), we chose those environmental variables with an increase of more than 50% of the mean square error (%IncMSE) – in the case of omission – (Fine_Sand_.g.kg_soil. and soil_temperature_10cm) together with the marked highest IncNodePurity-values (air_humidity, Clay_.g.kg_soil., forest_understory_.proportion. and dbh_standard_deviation_.cm.) for a subsequent model simplification based on Akaike information criterion (AIC; dredge function implemented in the MuMIn package).

**Fig A6. Variable selection based on a random forest for prediction of Shannon’s diversity.** The random forest method was used in order to determine those habitat variables that were more important to predict the Shannon’s diversity of the Silphidae taxon. This was performed by running regression trees in a high number (here, number of runs = 10^5^, cf. [1]). This method [2,3] will work even for highly correlated variables, allowing similar important scores for each. *A priori*, for a gaussian-GLMM (link = “log”, random effect at the plot level), we chose those environmental variables with an increase of more than 50% of the mean square error (%IncMSE) – in the case of omission – (mean_temperature, exploratory and soil_type) together with the marked highest IncNodePurity-value (stand_density_MTS_.trees.ha.) for a subsequent model simplification based on Akaike information criterion (AIC; dredge function implemented in the MuMIn package).

**Fig A7. Variable selection based on a random forest for prediction of Simpson’s dominance.** The random forest method was used in order to determine those habitat variables that were more important to predict the Simpson’s dominance of the Silphidae taxon. This was performed by running regression trees in a high number (here, number of runs = 10^5^, cf. [1]). This method [2,3] will work even for highly correlated variables, allowing similar important scores for each. *A priori*, for a gamma-GLMM (link = “log”, random effect at the plot level), we chose those environmental variables with an increase of more than 50% of the mean square error (%IncMSE) – in the case of omission – (soil_type and Fine_Silt_.g.kg_soil.) together with the marked highest IncNodePurity-values (exploratory and mean_temperature) for a subsequent model simplification based on Akaike information criterion (AIC; dredge function implemented in the MuMIn package).

REFERENCES

1. Lange M, Türke M, Pašalić E, Boch S, Hessenmöller D, Müller J, et al. Effects of forest management on ground-dwelling beetles (Coleoptera; Carabidae, Staphylinidae) in Central Europe are mainly mediated by changes in forest structure. For Ecol Manage. 2014;329: 166-176.

2. Breiman L. Random forests. Mach Learn. 2001;45: 5-32.

3. Prasad AM, Iverson LR, Liaw A. Newer classification and regression tree techniques: Bagging and random forests for ecological prediction. Ecosystems. 2006;9: 181-199.
