# Supplementary material for: Effects of abiotic environmental factors and land use on the diversity of carrion-visiting silphid beetles (Coleoptera: Silphidae): A large scale carrion study
Source: PLoS One. 2018 May 30;13(5):e0196839. doi: 10.1371/journal.pone.0196839 (PMC5976144; doi:10.1371/journal.pone.0196839)
Supplement: S2 File — (DOC) [file pone.0196839.s002.doc]

**Study site description.** Altitude increases from Schorfheide-Chorin (3 – 140 m above sea level (a. s. l.)) toward Hainich-Dün (285 – 550 m a. s. l.) and further to the Schwäbische Alb (460 – 860 m a. s. l.) [1]. The mean annual temperature is highest in Schorfheide-Chorin (8 °C – 8.5 °C) and decreases toward Hainich-Dün (6.5 °C – 8 °C) and further to the Schwäbische Alb (6 °C – 7 °C). Precipitation is highest in the Schwäbische Alb (700 mm – 1000 mm) and decreases toward Hainich-Dün (500 mm – 800 mm) and further to Schorfheide-Chorin (500 mm – 600 mm) [1]. In the Schwäbische Alb and Hainich-Dün, the age-class forests are dominated by European beech (*Fagus sylvatica*) and Norway spruce (*Picea abies*), whereas extensively used forests in the Schwäbische Alb are dominated by European beech (*F. sylvatica*). In Schorfheide-Chorin, age-class forests are dominated by Scots Pine (*Pinus sylvestris*) and European beech (*F. sylvatica*), whereas unmanaged forests are dominated by European beech (*F. sylvatica*). Cambisols are the main soil types in Schorfheide-Chorin, whereas Hainich-Dün plots are composed of Luvisols or Stagnosols. In the Schwäbische Alb region, Cambisols and Leptosols are the two predominant soil types (BExIS dataset 10580 version 2.7.6, EP_all_exploratories, Nieschulze & Schulze: https://www.bexis.uni-jena.de) [1].

**Fig A1. Example of an experimental plot.** The experimental plot No. 17 in the Schwäbische Alb region (AEW = Alb Experimental plot Wald (in English: forest)) is depicted. Inside the border of the plot chart, subplots associated with various projects are shown in different gray scales. Bold black lines outline skidder trails. The coordinates (2, 43) indicate an exposed piglet cadaver inside a wire cage. The coordinates (2, -43) indicate the respective control (only pitfall traps, no cadaver, and no wire cage) at a 100 m distance to the cadaver. Plot chart obtained from the BExIS platform (https://www.bexis.uni-jena.de) and modified after Jörg Hailer (contact person forest & hunting, plot maintenance & organization Schwäbische Alb).


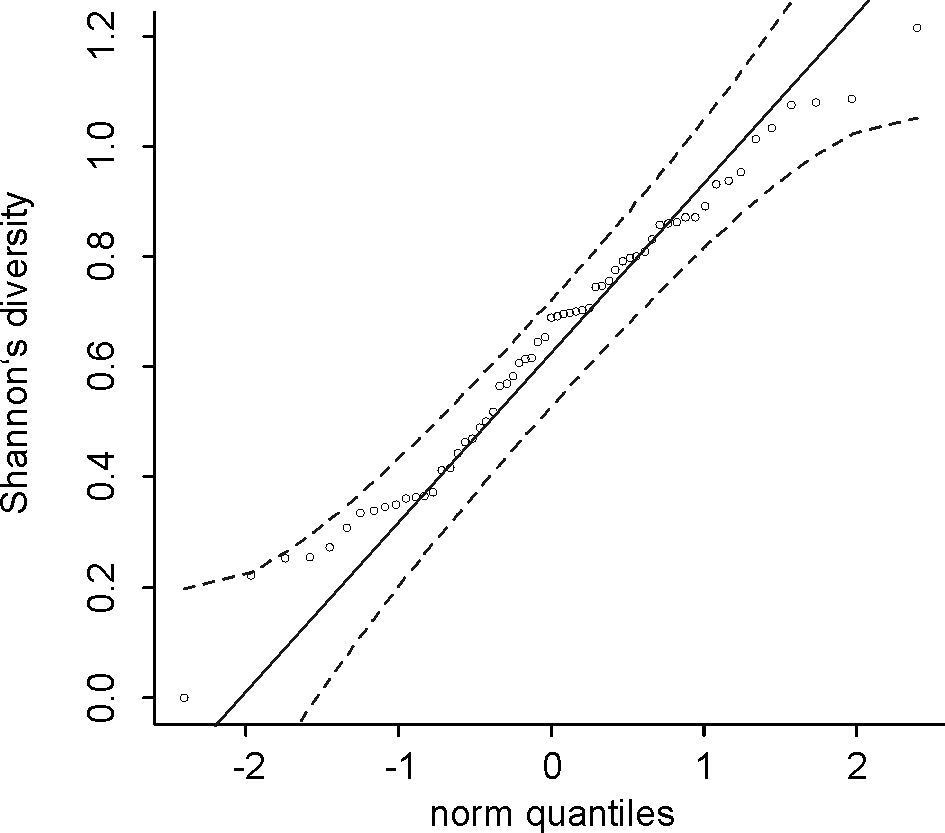


**Fig A2. Distribution plot of Shannon’s diversity of the silphid beetle taxon.** Shannon’s diversity shows a normal distribution (Shapiro-Wilk normality test, W = 0.98, P = 0.604).


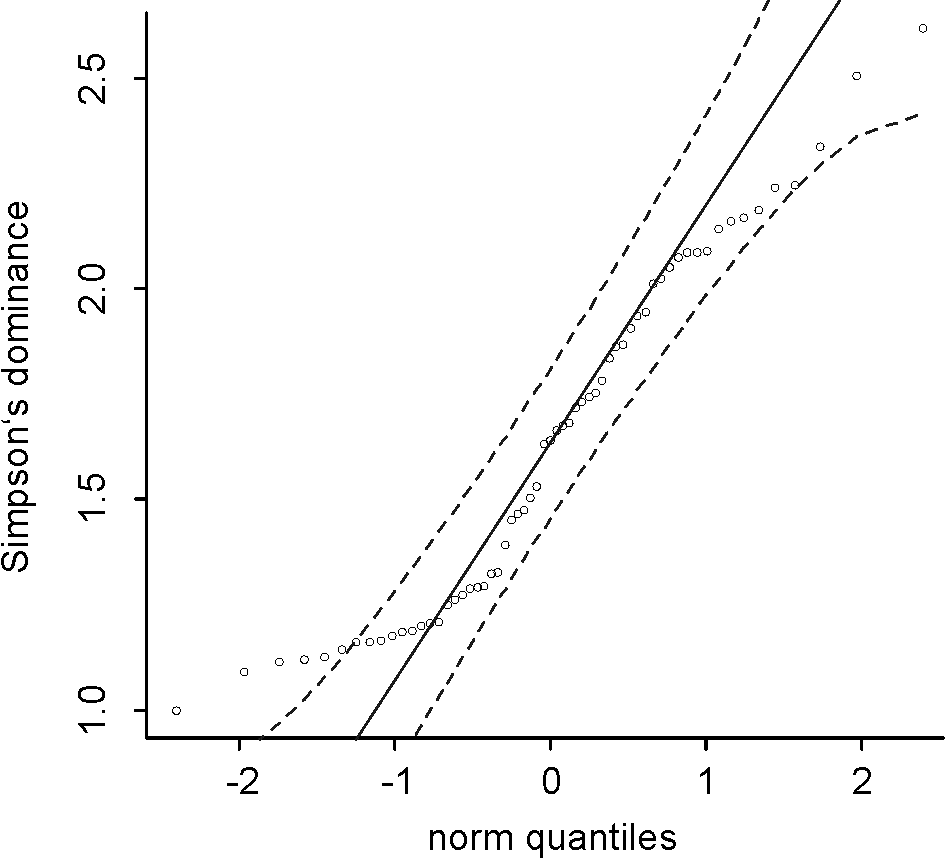


**Fig A3. Distribution plot of Simpson’s dominance of the silphid beetle taxon.** Simpson’s dominance shows a non-normal distribution (Shapiro-Wilk normality test, W = 0.94, P = 0.004).


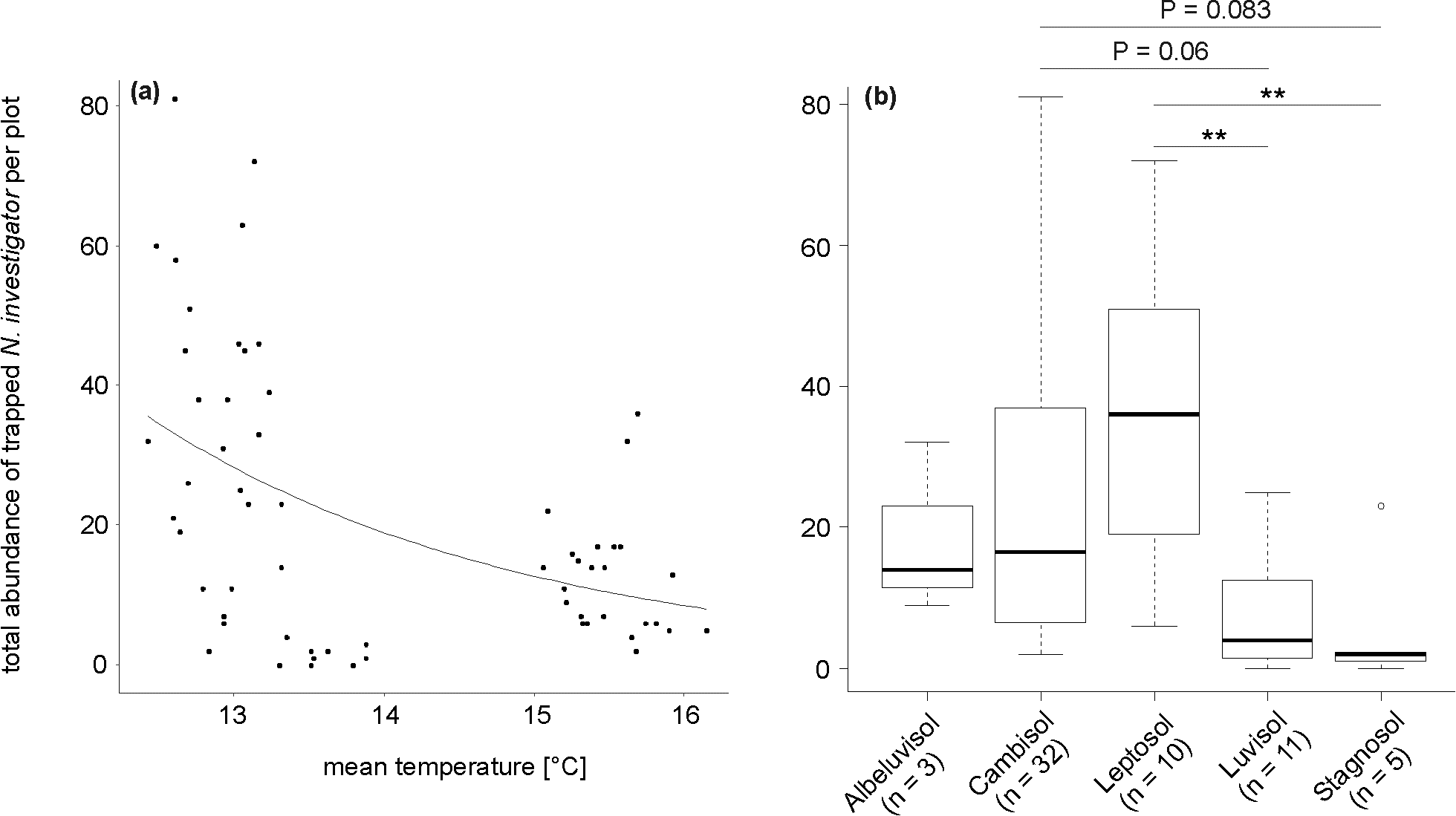


**Fig A4. Effects of environmental characteristics on *N. investigator* abundance.** Relationship between total abundance of trapped *N. investigator* per plot and **a** mean ambient temperature (F = 42.18, P < 0.001), **b** soil type (F = 13.01, P < 0.001). **a**: observed values (circles) and predicted values (connected by line) for the negative binomial-GLMM model (deviance = 56.44, P < 0.001), and **b**: box plot for the negative binomial-GLMM model (deviance = 56.44, P < 0.001) showing the median, the 75% percentile, the 25% percentile, the highest non-extreme value, the smallest non-extreme value, and the extreme values inside a category (Tukey tests, **P < 0.01). n = number of plots per soil type.


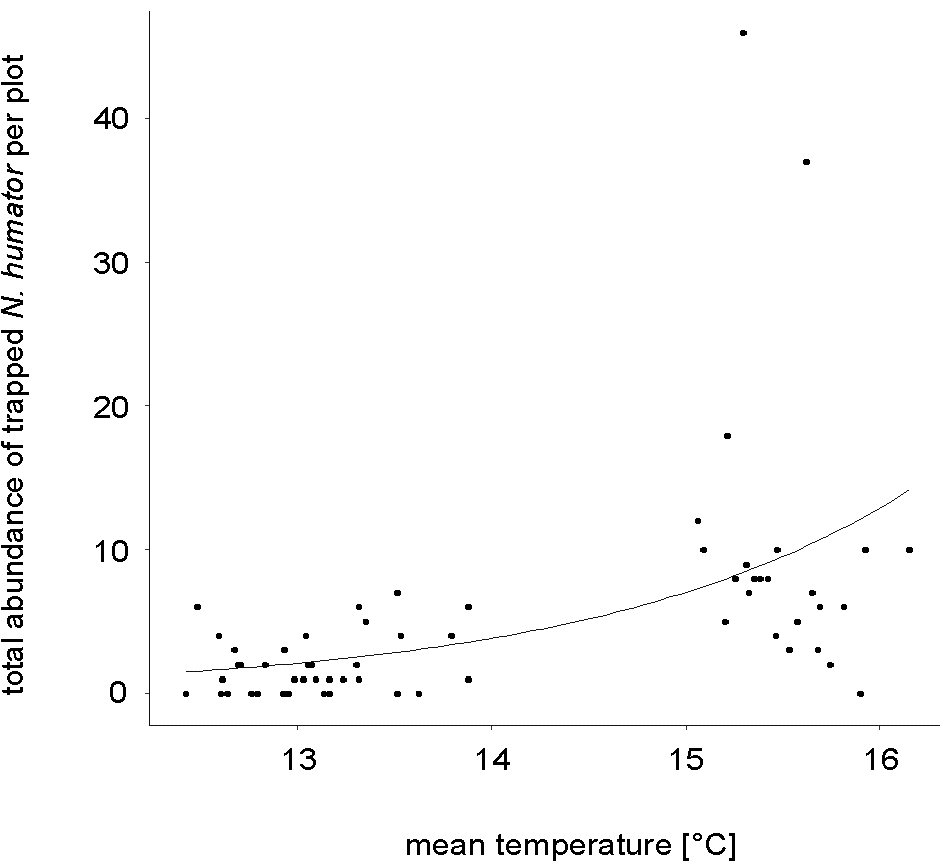


**Fig A5. Effect of ambient temperature on *N. humator* abundance.** Relationship between total abundance of trapped *N. humator* per plot and mean ambient temperature (F = 14.73, P < 0.001). Observed values (circles) and predicted values (connected by line) for the negative binomial-GLMM model (deviance = 55.73, P < 0.001).

REFERENCES

1. Fischer M, Bossdorf O, Gockel S, Hänsel F, Hemp A, Hessenmöller D, et al. Implementing large-scale and long-term functional biodiversity research: The Biodiversity Exploratories. Basic Appl Ecol. 2010;11: 473-485.
